# Statistical influence of travelling distance on home advantage over 57 years in the men’s German first soccer division

**DOI:** 10.1007/s12662-021-00787-7

**Published:** 2021-12-09

**Authors:** Nils Beckmann

**Affiliations:** Department of Electrical Engineering and Computer Science, Ostwestfalen-Lippe University of Applied Sciences and Arts, Campusallee 12, 32657 Lemgo, Germany

**Keywords:** Statistical analysis, Historic development, Geographical distance, Team performance, Home and away match results, Home advantage decline

## Abstract

A statistical analysis is presented that investigates the dependence of team cities’ geographical distances on the effect of home advantage (HA) for 57 years of the men’s German first soccer division (“Bundesliga”), including 17,376 matches (seasons starting from 1964 to 2020). The data shows that the HA can clearly be evidenced in the past and present (statistical *p‑value* < 0.01) and that it also exists for negligible distances (*p* < 0.01). The HA and the influence of distance on the HA both significantly decreased gradually over the last decades (*p* < 0.01). For the first and only time, the HA reversed into an away advantage (AA) for the season 2019/2020 (*p* < 0.01). The influence of distance on HA has been significant (*p* < 0.01) in the past (before about 1990) and contributed roughly by about half, compared to a situation without HA or AA. It increases with distance and saturates at around 100 km. Such saturation behaviour is in line with results from higher divisions of other countries with similar travelling distances such as Italy, Turkey and England. However, the distance-dependent contribution to HA has been approximately halved and reduced to an insignificant amount today. Furthermore, the temporal HA reduction is significantly larger for large distances compared to short distances (*p* < 0.01). Reporting and quantifying a reduction (*p* < 0.01) of the distance-dependent contribution to HA over a time span of 57 years is novel.

## Introduction

The home advantage (HA) is a well-known phenomenon in soccer worldwide (Carmichael and Thomas, 2005; Legaz-Arrese, Moliner-Urdiales, & Munguía-Izquierdo, 2012; Pollard and Pollard, 2005a; Pollard, 2006). Teams usually perform better if they play in their home city/stadium. The HA can be measured according to various factors such as match result or points won, goals and goals against, or via more specific metrics such as shot count or referee decisions (fouls and cards). It differs between countries (Nevill and Holder, 1999) and divisions (Leite and Pollard, 2018) and comprises a wide range of values. While in, e.g. Nigeria or Bosnia-Herzegovina (from 2006 to 2012), more than 70% of all points were gained playing at home (Pollard and Gómez, 2013), the HA can even reverse in isolated cases into an away advantage (AA) under special circumstances, e.g. for Spain and Germany during the onset of the COVID-19 pandemic in 2019 (Sánchez and Lavin, 2020).

Several underlying causes exist for HA, with varying and interacting effects (Pollard, 2008; Legaz-Arrese et al., 2012; Nevill and Holder, 1999). The main causes suggested included crowd size and effects (Johnston, 2008) such as (noise-induced) referee bias (Johnston, 2008; Wunderlich, Weigelt, Rein, & Memmert, 2021) or encouragement of players (Nevill, Newell, & Galec, 1996; Ponzo and Scoppa, 2014). These could be contrasted in detail, for example, due to the occurrence of matches in the absence of spectators during the COVID-19 pandemic from 2019 onwards (Fischer & Haucap, 2020; Sánchez & Lavin, 2020; Sors, Grassi, Agostini, & Murgia, 2020; Wunderlich et al., 2021). Further influences comprise travel and distance (Brown et al., 2002; Clarke & Norman, 1995; Goumas, 2014; Pollard, Silva, & Medeiros, 2008), familiarity with home conditions (Pollard & Pollard, 2005b; Pollard, 2002), stadium structure (Armatas & Pollard, 2014), territoriality (Pollard & Seckin, 2007; Neave & Wolfson, 2003; Pollard & Gómez, 2009), rule factors (Jacklin, 2005; Thomas, Reeves, & Davies, 2004), professionalism of players (Wolfson & Neave, 2004; Leite & Pollard, 2018), civil war or corruption (Pollard & Gómez, 2013) and gender (Pollard & Gómez, 2014), among others (Van Damme & Baert, 2019). One key challenge is to separate the respective quantitative contributions of the underlying influences (Carron, Loughhead, & Bray, 2005).

Travel is of particular interest, since all teams have to deal with this factor and can vary its conditions. The influence of travel and distance on soccer HA has been investigated more closely in many countries worldwide (Clarke & Norman, 1995; Goumas, 2014; Oberhofer, Philippovich, & Winner, 2009; Pollard, 1986, 2006; Pollard et al., 2008; Pollard & Seckin, 2007; Seckin & Pollard, 2007; Thomas et al., 2004), as well as for matches from the European UEFA Champions League, UEFA Europa League (Van Damme & Baert, 2019) and FIFA World Cups (Brown et al., 2002).

Results on travel distance vary depending on the setting and suggest no influence (Van Damme & Baert, 2019), for example explained by the comparably short distances within Greece (matches from 1994 to 2010) (Armatas & Pollard, 2014), minor influence (Pollard, 1986; Johnston, 2008; Pollard et al., 2008), for example reinforced by less overnight stays during World Cups (matches from 1987 to 1998) (Brown et al., 2002), major influence in vast countries such as Australia (Goumas, 2014) or influence that is simply present in Germany (1986–2010) (Oberhofer et al., 2009) or England (1970–1990) (Pollard, 1986; Clarke & Norman, 1995). The HA can be unusually high specifically in remote locations (Pollard & Seckin, 2007; Pollard & Gómez, 2013), such as in Brazil (Pollard et al., 2008) or Turkey (Seckin & Pollard, 2007), pronounced in specific regions such as the European Balkan (Pollard & Seckin, 2007) and lower in local derbies (Seckin & Pollard, 2007; Pollard, 1986, 2002), which may be explained by familiarity with home conditions if two teams share the same stadium, such as for example Ponzo and Scoppa (2014) in Italy. Further explanations regarding these travel effects comprise travel fatigue (Pollard & Pollard, 2005a; Leite & Pollard, 2018) promoted by remoteness and jet lag for long distances (Goumas, 2014), climate differences (Pollard et al., 2008), the number of fans that travelled with the away team (Pollard, 1986; Ponzo & Scoppa, 2014) as well as geographical and cultural isolation (along with remoteness) strengthening territoriality (Seckin & Pollard, 2007; Pollard & Seckin, 2007; Pollard & Gómez, 2009), the latter indirectly measurable via testosterone levels (Neave & Wolfson, 2003). Improved travel comfort nowadays (Pollard & Pollard, 2005a, b; Pollard, 1986) has been assumed to be a distance-dependent reason for HA reductions, for example in England (Thomas et al., 2004). Therefore, newer studies might find only less influence due to distance, such as that found by Van Damme and Baert (2019). However, the authors limited themselves only to top-class UEFA matches, where detriments (such as travel or adverse psychological influences of players) are more likely to be eliminated anyway through financial capacities, improved travel strategies (such as overnight stays) and more professionally trained players and referees (Wolfson & Neave, 2004; Stolen, Chamari, Castagna, & Wisloff, 2005; Pollard & Gómez, 2014). Appropriately, Leite and Pollard (2018) found a stronger influence for distance in lower divisions worldwide with less professional facilities.

To conclude, multifactorial explanations exist (Pollard, 2008; Pollard & Pollard, 2005a), and the relative (qualitative and quantitative) contributions of the mentioned factors are still to be established (Carron et al., 2005). Some of these are indirectly linked to distance (territoriality, familiarity with stadium conditions, number of fans travelling to the match, cultural differences, etc.), thereby imposing an indirect distance-dependent influence on HA (Van Damme & Baert, 2019). However, no previous study has explicitly assessed the change of distance-dependent influences on HA over time. Thus, the aim of this paper is to look only at the influence of distance on HA in the men’s German first soccer division “Bundesliga” and evaluate its change over time quantitatively. This league offers a large body of data since its start in 1964 in combination with decent travelling distances of up to about 800 km, which makes it a suitable candidate for such an analysis. Where possible, results are discussed in the context of other countries.

## Methods

The data used in this article are freely available in the public domain. Thus, no statement of ethical approval is required.

Match results of the men’s German first soccer division from years 1964 to 2020 (denoting the year in which the respective season started) were taken from online databases (Fussballdaten, 2015; DFB, 2021) with a total of 56 ∙ 306 + 1 ∙ 240 (in 1964) = 17,376 matches played by 55 teams. Each game with index *i* is assigned a normalised “result” value *r*_*i*_ in [0;1], which is set according to the match outcome (*r*_*i*_ = 0 (loss), *r*_*i*_ = 0.5 (draw), *r*_*i*_ = 1 (win)) for the respective team and indicating its performance/ability. This way, artificial mathematical alterations are avoided (Clarke & Norman, 1995) due to a change from 2 to 3 points per victory, which occurred for the German first division between 1994 and 1995. For comparison, for the 2‑point counting system, the normalised result value *r* directly translates into the percentage of points gained at home *g* (with respect to all points gained), which is a common measure in the literature. A mathematical analysis reveals that here *g* is usually slightly larger than *r* on average by an offset of *Δ*_*3P*_ := <*g*> − <*r*> ≈ + 0.01 = 1% when changing to a 3-point counting system (see Discussion for details). The average team performance or result for a specific ensemble of *N* matches is marked as *r(t)* or *r(d) *(*g*(*t*) or *g*(*d*) alike), as a function of time *t* or distance *d*, respectively. Averaging of *r* runs over all *N* match results of all home teams, neglecting the respective result for the away team. Therefore, an averaged value of *r* = 0.5 would indicate that there is no HA and a value of *r* = 1 would mean that the home team would always win (for *r* = 0 the HA is reversed and the away team always wins). Thus, the HA is larger with increasing *r*. Consequently, the HA will be regarded with respect to 0.5 and the term *r* −0.5 is used for analysis.

The respective geographical latitude *φ* and longitude *λ* coordinates of the cities of the teams in Germany are taken from another online database (Geonames, 2016). The respective absolute (travelling) distance | *d*| between two cities (indices 1 and 2) is calculated by the mathematical distance on a great circle via

1 $$| d| =R_{E}\cdot \cos ^{-1}(\delta )$$

where *R*_*E*_ = 6371 km is the average earth’s radius (Keller, 2003) and

2 $$\delta =\sin (\phi _{1})\cdot \sin (\phi _{2})+\cos (\phi _{1})\cdot \cos (\phi _{2})\cdot \cos (\lambda _{2}-\lambda _{1})$$

assuming thereby that the earth resembles a mathematically perfect sphere.

The average result *r* is calculated as a function of time *t* as *r(t)* (here, the starting year of the soccer season) and as a function of the distance *d* as *r(d)*. The applied discretisations for evaluation are *Δt* = 1 a (1 year) in time and *Δd* = 20 km in distance. This means that a data point *r(d)* contains match results from the semi-open interval [*d*; *d* + *Δd*]. All data points are provided with error bars, which denote the standard error confidence interval s = *σ* / √*N*, where *σ* is the standard deviation of *r* and *N* is the number of considered matches for the respective data point. The discretisation is necessary to ensure satisfying large enough match counts *N(d)* or *N(t)* per datum.

The software Gnuplot is used for diagrams and fits. It applies the Levenberg–Marquardt algorithm (Levenberg, 1944; Marquardt, 1963), here with weighting of the data points by (the inverse of) their respective error confidence. The related parameter errors are given as asymptotic standard errors s from the fit, which are used here for the calculation of statistical significance (given for statistical *p‑values* < 0.05) via *t*-test to check whether defective parameters are distinguishable. Bivariate Pearson correlation coefficients *c* in the closed interval [−1;+1] are calculated and Gaussian error propagation is applied.

## Results

**Fig. 1:**
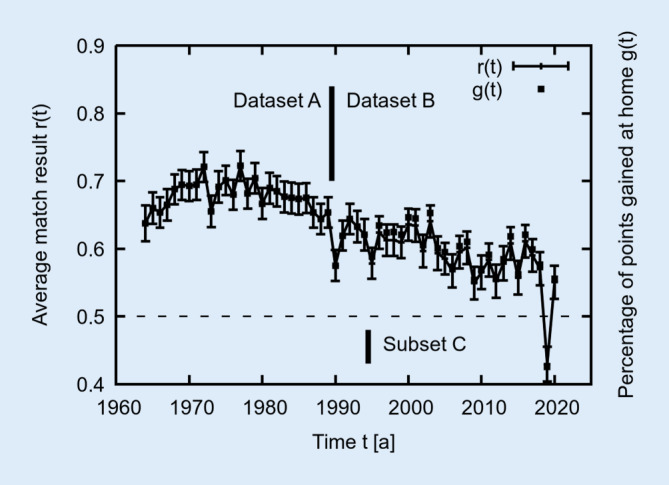
Dependence of the averaged result *r(t)* from the starting year *t* of the respective soccer season. Only home games of all competing teams are considered here. Each season *t* contains *N(t)* = 2 ∙ *m* ∙ (*m* − 1) / 2 = 306 matches with a constant of *m* = 18 active teams (except for 1964 with 240 matches where m = 16 teams). As a visual guide the 57 data points are connected with *lines*. The *vertical thick black line* between 1989 and 1990 denotes the dividing line for the respective datasets A and B analysed in Fig. 2. *Error bars* denote the confidence interval s = *σ* / √*N(t)*. In 1994 and before, the percentage of points gained at home *g*(*t*) is exactly equal to *r*(*t*). Starting from 1995 (see dataset C defined as a subset of dataset B), *g*(*t*) is usually marginally (less than 1%) larger than *r*(*t*) due to a change from a 2-point counting system to a 3-point counting system (see text)

The per-year averaged performance *r(t)* for all home games of the m = 18 active teams in the respective season (only in 1964, m = 16 teams) is shown in Fig. 1. The overall averaged home game result <*r*> = 0.631 ± 0.003 (<*g*> = 63.4%) is clearly distinct from 0.5; therefore an HA is present (Pollard & Pollard, 2005a). However, with progressing time *t*, one can see that the HA is decreasing by trend (*r(t)* is decreasing with a high anti-correlation of *c* = −0.82): In the 1970s roughly *r(t)* ≈ 0.7 (about 70% of all points gained at home) and from the 1990s onwards *r(t)* ≈ 0.6. In order to investigate the two regimes separately, two time ensembles are defined: From 1964 to 1989 (dataset A with <*r*_*A*_> = 0.679 ± 0.004 and 7890 matches with *c* = −0.10 and <*r*_*A*_> = <*g*_*A*_> = 67.9%) and thereafter from 1990 to 2020 (dataset B with <*r*_*B*_> = 0.591 ± 0.004 and 9486 matches with *c* = −0.59 and <*r*_*B*_> ≈ <*g*_*B*_> = 59.8%). They are isolated by a vertical black line in Fig. 1, where *r(t)* drops to 0.65 and below. This way, the majority of data points *r(t)* of A lie above B and both datasets can be analysed separately.

**Fig. 2:**
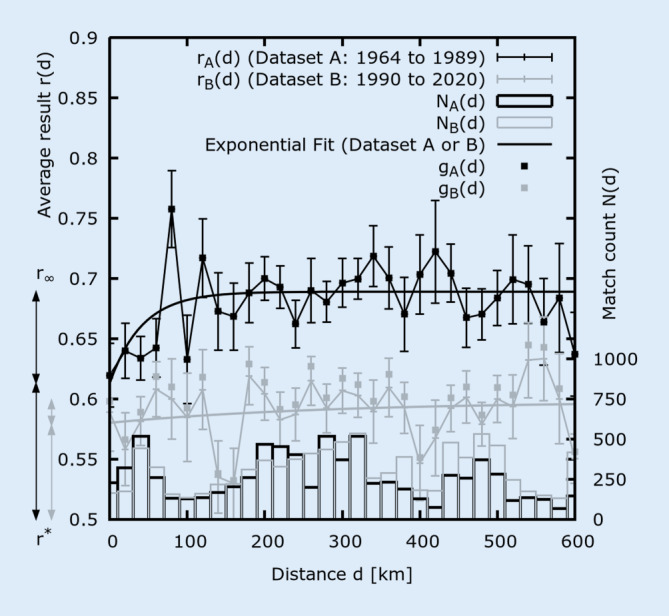
Dependence of the averaged result *r(d)* from the geographical distance *d* between the cities of the competing teams. As a visual guide the 31 data points are connected with *lines*. Two exponential fits to *r*(*d*) (one for each dataset) via Eq. 3 and parameters from Table 1 are drawn as *dotted lines*. According to our calculations, the maximum distance for two teams is *d* = 740 km (between the cities of Rostock and Freiburg). However, performance values for *d* > 600 km have been collapsed at *r*(600 km) in order to ensure a satisfying number of matches* N(d)* (shown as *solid blocks* at the *bottom*) for all considered data points. *Error bars* denote the confidence interval s = σ / √*N(d)*. For comparison, the percentage of points gained at home *g*(*d*) is depicted. This is equal to *r*_*A*_(*d*) but deviates marginally from *r*_*B*_(*d*)

Discretised plots of the averaged performances *r(d)* as a function of the cities’ distance *d* is shown in Fig. 2. Respective average distances per match are <*d*_*A*_> = (282 ± 163) km and <*d*_*B*_> = (325 ± 169) km (s ≈ ± 2 km, respectively). One observes that the HA is larger for dataset A (higher *r*(*d*)) compared to dataset B for the majority of distances *d* and *r*(*d*) decreases slightly for small *d*. A saturation of *r*(*d*) can be spotted around 100 km (for dataset A), but generally, *r*(*d*) is comparatively constant. A linear fit (not shown) for data points *d* ≥ 100 km to each dataset separately reveals that the slopes cannot be unambiguously classified as positive or negative according to error (both slopes << 0.01 per 100 km). This finding is also in line with calculated correlation coefficients *c* (of distance *d* vs. result *r*) to be around zero for both datasets (0 ≈ | *c*_*A/B*_ (*d* ≥ 100 km) | << 0.01). Still, for both datasets *r*(0 km) > 0.5 is clearly valid, which indicates that a negligible distance *d* is still connected with a detectable HA. Nevertheless, as mentioned above, a slight decrease in the HA of the home team can be observed for short distances *d*, especially for dataset A. Accordingly, for d ≤ 100 km small positive correlations of (A) 0.070 (*p* < 0.01) and (B) 0.021 (insignificant) as well as positive linear slopes of (A) 0.071 and (B) 0.020 (each per 100 km) can be found in this region. In order to investigate the influence of distance more closely, an exponential function according to Eq. 3 has been fitted to both datasets separately

3 $$r\left(d\right)=r_{0}+r_\infty\cdot e^{-\frac{d}{d_{0}}}$$

where the fit parameter *r*_*0*_ represents the maximal result value *r*(*d*) and thus maximal HA and* r*_*∞*_ (< 0) and *d*_*0*_ (> 0 km) represent the distance-dependent contribution and influence on HA. The motivation for an exponential saturating function is that the average performance *r*(*d*) first increases, but then seems to stabilize for large distances *d*, as pointed out before. The results of the least square fit procedure are shown in Table 1.

**Table 1:** Fit parameters of Eq. 3 for the respective dataset curves displayed in Fig. 2 and according continuative calculations

Fit parameter	Dataset A (1964–1989)	Dataset B (1990–2020)	Explanation of parameters in Eq. 3
*r*_*0*_	0.689 ± 0.006 (±0.9%) ***	0.598 ± 0.029 (±4.8%) ***	Maximal result value *r*(*d*) and thus maximal HA
*r*_*∞*_	−0.076 ± 0.026 (±34%) **	−0.018 ± 0.025 (±139%)	The distance-dependent contribution to HA (in contrast to r*)
*d*_*0*_	(41 ± 27) km (±66%)	(283 ± 1131) km (±400%)	A saturation distance for the HA
*Calculation*			
*r*(0 km) = *r*_*0*_ + *r*_*∞*_	0.613 ± 0.032 (±5.2%) ***	0.580 ± 0.054 (±9.3%) ***	Maximal distance-independent HA for 0 km travelling distance
r* := *r*(0 km) − 0.5	0.113 ± 0.032 (±28%) **	0.080 ± 0.054 (±68%)	Distance-independent contribution to HA (in contrast to *r*_*∞*_)
*r*_*HA*_ := *r*_*0*_ − 0.5 = |r*|+|*r*_*∞*_|	0.189 ± 0.006 (±3.1%) ***	0.098 ± 0.029 (±30%) **	Combined HA (distance-dependent and distance-independent)
*α* := |*r*_*∞*_ / *r*_*0*_|	(11.0 ± 3.8) % (±35%) **	(3.0 ± 4.3) % (±144%)	Relative share of the distance-dependent contribution on the maximal HA (*α*) and on the combined HA (*β*)
*β* := |*r*_*∞*_ / *r*_*HA*_|	(40 ± 15) % (±38%) *	(18 ± 31) % (±172%)	

The given errors are the asymptotic standard errors s according to the least square fit procedure. The rightmost bracket gives the relative error in percent (%). The stars (*) denote that the respective value is significantly different from zero (*p < 0.05, **p < 0.01, ***p < 0.001). The values of all calculations for datasets A and B are significantly different (*p* < 0.01), except for the *d*_*0*_ parameter. These differences represent a significant change (decrease) of home advantage (HA) over the decades. The fit parameter *r*_*0*_ represents the maximal result value *r*(*d*) in the closed interval [0;1] regarding normalised match results (0 = loss, 0.5 = draw, 1 = win). Thus, the more *r*_*0*_ exceeds the value of 0.5 (or *r*_*HA*_ the value of 0.0), the more HA is present. Furthermore, since *r*(0 km) > 0.5 is valid (or r* > 0), an HA also exists for negligible travelling distances, for example in local stadium derbies (*d* ≈ 0 km). The fit parameter *r*_*∞*_ represents the distance-dependent contribution to HA, which can be contrasted to the distance-independent contribution r*. The parameter *d*_*0*_ is a measure for the travelling distance above which HA saturates (exponentially according to the model of Eq. 3). The variables *α* and especially *β* show the relative shares of the distance-dependent contribution (*r*_*∞*_) to the maximal result or HA (see *α*) as well as to the combined HA (see *β*), directly displaying the maximum possible impact of travelling distance on the normalized result or HA. A robustness test for this analysis with normalised result value has been executed relating to an analysis with 3 points per win (see Discussion)

## Discussion

First, it is notable that *r*(0 km) = *r*_*0*_ + *r*_*∞*_ >0.5 (*p* < 0.01), which means that the HA cannot be solely explained by the geographical/travelling distance *d* alone, as reported before (e.g. Carmichael & Thomas, 2005; Pollard, 2006, 2008; Legaz-Arrese et al., 2012). The HA is predominantly present, even if teams with minimal distance *d* (e.g. if they belong to the same city) play each other (Clarke & Norman, 1995; Ponzo & Scoppa, 2014). Familiarity with home conditions (Pollard, 2002), territoriality (Wolfson & Neave, 2004) or crowd effects (Johnston, 2008; Nevill et al., 1996) among other factors can cause this finding. However, this distance-independent contribution to HA has significantly (*p* < 0.01) decreased over time (see r* of datasets A and B in Table 1).

Since *r*_*∞*_ is negative throughout and non-zero (*p* < 0.01 for dataset A), an influence of the distance *d* on the HA is present (see Eq. 3). However, it also has significantly (*p* < 0.01) decreased (*r*_*∞*__,B_ < *r*_*∞,A*_). On the one hand, shorter distances *d* reduce the HA of the home team and on the other hand, the HA saturates for larger distances *d*. The total influence of distance is smaller compared to the distance-independent influences on HA, since |*r*_*∞*_*|* < |r*| is significant (*p* < 0.01) in the past (A) and the present (B). The results suggest that up to half (*p* < 0.01) of the total HA has been explicable by distance-related effects in the past (see *β*_*A*_ in Table 1), while these effects have been roughly halved to an insignificant amount nowadays (see *β*_*B*_).

These findings will be discussed in the context of selected other countries. Pollard (1986) analysed the two highest divisions in England (1970–1981), a country with distances comparable to Germany, where he found lower but existing HA in London local derbies (394 matches) and no further influence of travel distance above 200 km (3496 London-related matches and 6274 others). These results are similar to the saturation and drop in HA for shorter distances reported here. Using Eq. 3 one might derive *r*_*∞*_ = −0.082 (*r*_*0*_ = 0.643) from the dataset he used, coinciding with the distance-dependent influence *r*_*∞*_= −0.076 ± 0.026 found here of dataset A (1964–1989) in Table 1. Appropriately, Clarke and Norman (1995) found an increase of HA with distance in the subsequent years (1981–1990) for the first soccer division in England. The HA reduction in local derbies has also been found in the Turkey Super League (Seckin & Pollard, 2007) from 1994 to 2005 (*r*_*∞*_ = −0.040 and *r*_*0*_ = 0.617 derived from the dataset they used) comparable with the value of *r*_*∞*_ = −0.018 ± 0.025 found here for dataset B (1990–2020). The same trend has been described for the Italian Seria A (Ponzo & Scoppa, 2014) from 1991 to 2012 (7398 matches) even between teams that share the same stadium (128 matches), probably cancelling out familiarity with home conditions as a factor in this case (Pollard, 2002). One limitation of the present study is that the number of same *stadium* derbies is unknown, which might reduce HA for the shortest distances (*d* ≈ 0 km). However, the number of same *city* derbies is only about 10% (40 of 389 matches with *d* < 20 km), thus playing a minor role here.

As a mathematical robustness test, we repeated our analysis for the percentage of points gained at home *g* with 3 (win), 1 (draw) and 0 (loss) points per match (3-point counting system), which is a common measure for many soccer divisions worldwide (e.g. in England since 1981, Turkey since 1987, Italy since 1993), including the German Bundesliga since 1995. As noted before, for the 2‑point counting system (1994 and earlier) the identity *r* = *g* holds. For the years from 1995 to 2020 (defined as dataset C as a subset of dataset B), we find very high correlations of *c* = 0.9997 between the two types of analysis for *r*_*C*_(*t*) with *g*_*C*_(*t*) as well as for *r*_*C*_(*d*) with *g*_*C*_(*d*). However, *g* is usually (except for the year 2019 with AA) slightly larger than the result value *r* by an offset of *Δ*_*3P*_ = + 0.0083 ± 0.0026 ≈ (0.8 ± 0.3) % ≈ 1% on average (<*g*_*C*_> = 0.594 ± 0.003 = 59.4% and <*r*_*C*_> = 0.586 ± 0.003 = 58.6%), which has to be taken into account. All according fit parameters for *g*(*d*) = *g*_*0*_ + *g*_*∞*_ ⋅ *exp*(−*d* / *d*_*0g*_) equivalent to Eq. 3 (*g*_*0*_ = 0.597 ± 0.011 = 59.7%, *g*_*∞*_ *=* *−*0.021 ± 0.026 = −2.1%, *d*_*0g*_ = (122 ± 313) km, g* = 0.076 ± 0.037 = 7.6%, *g*_*HA*_ = 0.097 ± 0.011 = 9.7%, *α*_*g*_ = (3.6 ± 4.4) %, *β*_*g*_ = (22 ± 29) %) coincide well with fit parameters of *r*(*d*) (*r*_*0*_ = 0.589 ± 0.009, *r*_*∞*_ = −0.020 ± 0.025, *d*_*0*_ = (111 ± 276) km, r* = 0.069 ± 0.034, *r*_*HA*_ = 0.089 ± 0.009, *α* = (3.4 ± 4.3) %, *β* = (22 ± 30) %) for dataset C and even with parameters of dataset B (1990–2020) within the margin of error (see Table 1 for comparison). As a mathematical consequence of this rule change, the percentage of points gained at home slightly increased statistically for all teams (up to *Δ*_*3P*_ ≈ 1%). Thus, teams with comparatively higher HA were marginally advantaged in gaining points at home (Jacklin, 2005; Clarke & Norman, 1995) but not regarding match results (outcome). Since the difference *Δ*_*3P*_ is small for this dataset, the main conclusions drawn from the match results *r* regarding HA are not affected and also apply for the points gained at home *g*. To note, *Δ*_*3P*_ usually increases with more points per victory as well as for higher HA or AA (for example, one finds *Δ*_*3P*_ *=* 1.6% for dataset A with higher HA, see Fig. 1) and thus might play a more relevant role for other studies concerning different divisions or sports.

Oberhofer et al. (2009) also analysed the German first division, but restricted themselves to years from 1986 to 2006 (6389 matches), which is partly comparable to dataset B here. The authors state that travelling is especially detrimental to away teams at short distances, which can be seen as another formulation for the drop in HA reported here (see Fig. 2). Further, they stated that the influence of distance over time (1986–2006) did not change significantly. Here, in contrast, the distance-dependent contribution *r*_*∞*_ significantly decreased (*p* < 0.01) from past (A) to present (B), indicating a reduction of the distance-dependent influence on HA. It is argued here that Oberhofer et al. (2009) did not detect this decrease, since they considered fewer seasons (only 21 compared to 57 here) and more recent seasons (which already exhibit less influence of distance). For the Superleague Greece (1994–2010), the influence of distance on HA has been reported to be insignificant, which has been explained by the specific and very short travelling distances in Greece (Armatas & Pollard, 2014). Correspondingly to the findings of our study, another reason might be their restriction to more recent years, as noted above. The distance-dependent contribution *r*_*∞*_ has decreased and lost significance over time (from *p* < 0.01 for A to insignificant for B).

For Brazil (Pollard et al., 2008), Australia (Goumas, 2014), Turkey (Seckin & Pollard, 2007) or the Balkan region (Pollard & Seckin, 2007) increases for the home team HA have been reported for the largest travelling distances of over 1000 and up to 5432 km (Goumas, 2014). These results have been attributed to climate conditions in different regions, time zones and jet lag, remoteness and ethnic or cultural differences. However, the largest distances for Germany are around 740 km (between the cities of Freiburg and Rostock), with low to no relevance of these mentioned factors. Accordingly, no such increases in HA for the largest distances have been found (see Fig. 2). However, to check for remoteness explicitly, the teams’ individual HA (calculated as the average result <*r*_*team,home*_> of home matches divided by the average result of all matches <*r*_*team*_> of the team to account for the different playing strengths of teams) has been compared with the average distance <*d*_*team*_> travelled (not shown). Indeed, there are no outliers for the largest distances <*d*_*team*_> and also slope (−0.006 per 100 km with s = ±260%) as well as correlation (*c* = −0.05) are even slightly negative and insignificant according to margin of error (the same is true for dataset A or B alone).

To summarise, the literature findings regarding the influence of distance on HA for the cited countries England (Pollard, 1986; Clarke & Norman, 1995), Germany (Oberhofer et al., 2009), Italy (Ponzo & Scoppa, 2014) and Turkey (Seckin & Pollard, 2007) are qualitatively comparable and understandable in context with the saturation behaviour proposed here (see Fig. 2). The abovementioned particular influences for large distances as suggested for Brazil (Pollard et al., 2008), Australia (Goumas, 2014), Turkey (Seckin & Pollard, 2007) or the Balkan region (Pollard & Seckin, 2007) do not play a role for Germany due to its limited extent. In possible contrast to Greece (Armatas & Pollard, 2014), significant influence of distance on HA has also existed in the past for the lowest distances; however, this has declined to an insignificant amount nowadays.

Looking at the underlying causes for the reduction of the distance-dependent influence detected here, altered balances in the distance-dependent factors travel fatigue (Pollard & Pollard, 2005a; Goumas, 2014) and away team fan support (Ponzo & Scoppa, 2014; Seckin & Pollard, 2007) have been suggested. For example, travel fatigue could have been reduced nowadays by less stressful travelling, more travel comfort (Pollard & Pollard, 2005a) or extended overnight stays (Brown et al., 2002) for relaxation, which might explain the reduction in distance-dependent HA over the decades found here. A travelling distance of around 100 km might have been a critical distance for notable travel fatigue in the past (see dataset A in Fig. 2) due to inferior travel facilities. Teams in Germany usually travel by bus to away matches, nowadays with notable comfort (Autobild, 2021), since various travel factors (bus size, seat width and comfort, travel speed and duration, vibration attenuation, paving quality, etc.) have improved over the decades (e.g. HOV, 2021; MAN, 2021). These long-term developments went along with increasing club budgets and Gross Domestic Product (GDP) of Germany (DeStatis, 2021). These financial and travelling possibilities are reduced in lower divisions, which consequently leads to higher HA (Leite & Pollard, 2018). In addition, general improvement of travel possibilities may also have increased accessibility for the away team’s fans to accompany their team in larger numbers. Thus, more fans of the away team would be present in the stadium, decreasing the home team’s HA via noise and crowd effects and thus altering the amount of important referee decisions (Carron et al., 2005; Nevill et al., 1996; Ponzo & Scoppa, 2014). However, the influence of the crowd on HA might not always be significant or relevant, as suggested by recent studies in the context of COVID-19 for Austria and England (Sánchez & Lavin, 2020) or other European countries (Wunderlich et al., 2021).

Furthermore, it is a well-known fact that intraspecific territorial aggression in vertebrates declines with distance up to a maximum distance from their territory centre (Myrberg & Thresher, 1974; Lorenz, 1966). Accordingly, the saturation of HA with distance found here may be linked to a reduction of the away team’s intraspecific aggression up to their (perceived) territory border, which could be around 100 km away from their home stadium. However, players as well as referees may act more professionally today, and are also better trained physiologically (Stolen et al., 2005) and psychologically. They may thus be less influenced by travelling, territorial influences, the surrounding of the playing field and crowd effects (e.g. noise), especially in higher divisions (Leite & Pollard, 2018). To train such behaviour has been proposed by Wolfson and Neave (2004) as a strategy for away team coaches (Pollard & Gómez, 2014). Thus, parts of the reductions of the distance-dependent as well as the distance-independent contributions to the HA may be due to developments that might be summarised as increased professionalism and internationalisation nowadays or, alternatively, altered idiosyncrasies of players (Thomas et al., 2004) and stadiums. Players might be less (emotionally) affiliated with a home stadium, location or city as well as less unnerved by foreign places and stadiums, since they more often change teams, clubs or places, or come from other countries, which might reduce familiarity with home conditions (Pollard, 1986) and territoriality (Wolfson & Neave, 2004; Neave & Wolfson, 2003). Accordingly, Pollard (2002) found that the HA is reduced when a team changes stadium. Thus, a change of territoriality over the decades may be an indirect cause (Thomas et al., 2004) for the reduction of the influence of distance here. To measure territoriality, salivary testosterone levels of players have been successfully used as an indirect marker, and it has been shown that testosterone levels are significantly higher before a home game than an away game (Neave & Wolfson, 2003). Indeed, there have been reports from European (Andersson et al., 2007; Carruthers, 2009) and North American countries (Travison, Araujo, O’Donnell, Kupelian, & McKinlay, 2007) that testosterone levels in men have been gradually decreasing for the last century in the general population overall. These findings could indicate a decline in territoriality, which could also be connected to a diminution of the distance-independent as well as the distance-dependent HA (e.g. if testosterone level differences between teams are correlated with distance according to intraspecific aggression).

Another factor is the increase of points per victory from 2 to 3, which had been identified as a main cause for the observed drop in HA in 1981 in soccer in England (regarding the ratio of numbers of home wins to away wins) by Jacklin (2005) due to lessened incentives of away teams to settle for a draw (Thomas et al., 2004). As noted above, this rule change *alone* imposes a mathematical increase of about 1% on the percentage of *points* gained at home *g*(*d*) (Clarke & Norman, 1995). In the men’s German first soccer division, this change happened between 1994 and 1995. However, this issue led only to a small drop of HA for a single year regarding normalised match result (*Δr* = −0.043 = −4.3%), while a decreasing trend of HA had already set in during the years before (see Fig. 1). This may indicate only a minor influence of this issue here. It is interesting to note that another larger drop of HA (*Δr* = −0.078 = −7.8%) is clearly visible in the year 1990, which is the year of German reunification. Never again after this year did the HA reach result values *r*(*t*) of 0.65 or above (see Fig. 1). The reunification also led to other socioeconomic alterations within Germany (e.g. Hesse et al., 2003) and might have reduced the sense of territoriality of the players (Wolfson & Neave, 2004) or accelerated the abovementioned internationalisation and professionalism within the soccer divisions. However, the largest drop of HA in a single year (*Δr* = −0.140 = −14%) in the whole history of the Bundesliga happened in 2019, reversing the HA into an AA for the first and only time (*r* = 0.430 < 0.5). In this year, the global pandemic of COVID-19 spread, also imposing social disruptions. During this time, many soccer matches worldwide (and also in Germany) were held without spectators. From the analysis of tens of thousands of those matches in European major and minor leagues, Wunderlich et al. (2021) found a significant reduction of referee bias and shots, which they attributed to omitted crowd effects such as crowd noise. However, they only found an insignificant lowering of HA, from which they concluded that there must be more important influences to HA than crowd effects alone. Similarly, Sors et al. (2020) claimed a disappearance of referee bias and HA in the absence of spectators for the two highest leagues in Germany, Spain, England and Italy in 2019. In contrast, Sánchez and Lavin (2020) found a change of HA (and also of AA) between playing with or without a crowd only for the former two countries (and no change of HA for the latter). Here, for comparison, spectators increased from 5.9 million in 1964 (about 25,000 per match) to about 13.3 million in 2018 (DFB, 2021). Due to COVID-related political decisions, spectators were reduced in 2019 down to about 9.1 million in 2019, when the aforementioned AA set in (in line with Sánchez & Lavin, 2020). In contrast, however, the HA recovered in 2020 (*r* = 0.551 > 0.5), even when spectators were reduced to less than 0.2 million. For the total dataset (1964–2020), we even find a high anti-correlation (*c* = −0.572 < 0 with *p* < 0.001) between *r*(*t*) and the number of spectators (not shown). Surprisingly, this is a hint that more spectators could even reduce HA, however superimposed by others covariates (Van Damme & Baert, 2019; Pollard & Gómez, 2013). Hence, the onset of the emerging crisis of COVID-19 with its accompanying socio-economic changes (rather than spectators or distance), possibly also provoking psychological effects, is correlated with the reduction (and reverse of) HA in 2019, as it has been shown that socioeconomic changes (such as crises and civil wars) may also influence the HA (Pollard & Gómez, 2013).

## Conclusions and implications

To conclude, the HA over the whole history of the men’s German first soccer division “Bundesliga” (for 57 years from 1964 to 2020) and its dependence on geographical (travelling) distance has been investigated. The HA is clearly present (*p* < 0.01), but its distance-dependent and distance-independent contribution both decreased over the decades (*p* < 0.01). This is the first time that a reduction in the distance-dependent HA is reported for Germany (Oberhofer et al., 2009). The HA increases with distance, but saturates for distances around 100 km, which is qualitatively comparable to findings for other countries with similar travelling distances, especially England (Pollard, 1986; Clarke & Norman, 1995), Italy (Ponzo & Scoppa, 2014) and Turkey (Seckin & Pollard, 2007).

Factors that might explain the reductions of the distance-dependent and distance-independent influence on HA have been discussed. These include improved travel conditions (and strategies) nowadays reducing travel fatigue (Pollard & Pollard, 2005a; Thomas et al., 2004; Van Damme & Baert, 2019), larger numbers of fans that travelled with the away team (Nevill et al., 1996; Ponzo & Scoppa, 2014; Seckin & Pollard, 2007), reduced familiarity and territoriality due to more internationalisation and professionalism nowadays (Stolen et al., 2005; Wolfson & Neave, 2004; Neave & Wolfson, 2003) in combination with declined testosterone levels on a population level (Andersson et al., 2007; Travison et al., 2007; Carruthers, 2009) as well as distance-dependent intraspecific aggression (Myrberg & Thresher, 1974; Lorenz, 1966). Selected individual larger drops in HA from one season to another (and even the occurrence of an AA in 2019) coincide with profound socioeconomic changes (Hesse et al., 2003) or rule changes (regarding points per victory), but tend to be uncorrelated with travelling distances or numbers of spectators (Wunderlich et al., 2021).

Following earlier reports (Nevill et al., 1996; Nevill, Balmer, & Williams, 2002; Ponzo & Scoppa, 2014; Wunderlich et al., 2021; Sors et al., 2020), the number of referee decisions for the respective team can depend on attendance, crowd noise and fan balance. If, for example, away team fan attendance (arriving from remote locations) is a distance-dependent contributor, one could investigate whether this correlates with travelling distance and subsequently with the number of referee decisions in the match. Besides, for specific travel conditions (Armatas & Pollard, 2014) or in lower divisions (Leite & Pollard, 2018) with very short distances between cities and lower attendance, different influences of distance may be obtained (Clarke & Norman, 1995; Pollard, 1986, 2008; Whatling, Micklewrith, & Griffin, 2012). An approximation has been made in this paper: The distance *d* on a perfect sphere is slightly different from the real geographical distance or travelling distance or travelling time. These quantities as well as direct measurements of the away team’s travel fatigue (Pollard & Pollard, 2005a) and testosterone levels (Neave & Wolfson, 2003) depending on distance may yield more precise measures of the variation of the teams’ performance. Although influence of distance on a team’s performance has declined over the decades, it is still notable today for coaches to consider that distance and travel are factors in the team’s playing performance. However, for a complete understanding of causes for HA, further research is necessary. Time-dependent datasets over decades on factors such as referee decisions (Ponzo & Scoppa, 2014; Wunderlich et al., 2021; Sors et al., 2020), fans that travelled with the away team (Ponzo & Scoppa, 2014) depending on distance, team budget and coaching time invested per player (professionalism), frequencies of club changes of players (internationalisation), crowd noise (Carron et al., 2005; Sors et al., 2020), mental and physiological state of players (Stolen et al., 2005) including testosterone levels (Andersson et al., 2007; Neave & Wolfson, 2003), result datasets of other (lower) divisions from Germany as well as other countries (and so on) could be of use for future analysis and comparison.

## Declarations

For this article no studies with human participants or animals were performed by any of the authors. All studies performed were in accordance with the ethical standards indicated in each case.
